# Influence of Environmental Factors and Microstructure on Water Vapor Permeability of Gypsum-Based Inorganic Mineral Materials

**DOI:** 10.3390/ma19194240

**Published:** 2026-10-07

**Authors:** Lu Bai, Jingchao Xie, Yue Xie

**Affiliations:** 1 School of Civil Engineering, Inner Mongolia University of Technology, Hohhot 010051, China; 2 Inner Mongolia Key Laboratory of Green Consruction and Ineligent Operation and Maintenance of Civil Engineering, Inner Mongolia University of Technology, Hohhot 010051, China; 3 School of Civil Engineering and Architecture, Beijing University of Technology, Beijing 100124, China

**Keywords:** gypsum-based materials, water vapor permeability, relative humidity, microstructure

## Abstract

**Highlights:**

In this study, the water vapor permeability of gypsum-based inorganic mineral materials was systematically investigated using the cup method. The effects of the relative humidity (RH) gradient and average RH on the total vapor permeability were quantitatively analyzed to elucidate the governing factors of vapor transport behavior. Combined with the preceding sorption analyses and microstructural characterization, the underlying mechanisms of water vapor transport properties of gypsum-based inorganic mineral materials were interpreted from a microstructural perspective.

**What are the main findings?**

- Water vapor permeability is primarily governed by the average relative humidity, and the effect of the RH gradient is comparatively minor under the tested conditions.
- Permeability varies nonlinearly with RH, well fitted by a binomial function. It is nearly constant below 70% RH but drops sharply above 70% RH.
- G-D’s well-connected meso/macroporous channels yield the highest permeability, while G-A’s dense interlocking structure with few connected pores gives the strongest vapor resistance.

**What are the implications of the main findings?**

- These findings provide a quantitative and mechanistic understanding of water vapor transport in gypsum-based materials.
- The results can serve as a theoretical basis for optimizing the design and application of humidity-regulating gypsum composites.

**Abstract:**

The water vapor permeability coefficient is a key parameter describing moisture transport in building materials, exerting a crucial influence on their hygrothermal performance and indoor humidity regulation. However, uncertainties remain in existing experimental methods and parameter-correction approaches. In this study, three gypsum-based inorganic materials—gypsum–zeolite (G-Z), gypsum–diatomite (G-D), and gypsum–magnesium aluminum silicate (G-A)—were systematically investigated using the wet-cup method to measure their vapor permeability coefficients. The effects of the average relative humidity (RH) and RH gradient on the measured results were quantitatively analyzed. The water vapor transport characteristics of gypsum-based inorganic mineral materials were also analyzed. The results indicate that the influence of the RH gradient is comparatively minor, whereas the average RH is the dominant factor. Within the average RH range of 22–90%, the vapor permeability coefficients of the three materials exhibit a nonlinear dependence that can be accurately described by a binomial function. In the low-humidity range (RH < 70%), the coefficients remain nearly constant, while in the high-humidity range (RH > 70%), they decrease significantly with increasing RH. Microstructural characterization further reveals that G-Z exhibits a well-developed microporous structure and large specific surface area, leading to high moisture adsorption but low permeability; G-D shows superior permeability due to its well-connected meso-and macroporous channels; and G-A forms a dense interlocking structure with few connected pores, resulting in the highest vapor resistance among the three materials.

## 1. Introduction

In modern buildings, interior finishing materials serve not only as a decorative layer but also as a critical component that influences the overall building’s environmental performance. Their hygroscopic properties directly affect coupled heat and moisture transfer within building envelopes, and their moisture sorption and transfer capacities govern the buffering effect on indoor humidity fluctuations. As the “first contact layer” between the indoor environment and the building envelope, interior finishes thus play a vital role in research on indoor environment regulation, energy-efficient design, healthy buildings, and nearly zero-energy buildings.

Interior finishing layers are directly exposed to the indoor environment, where temperature generally remains relatively constant, typically varying within approximately 15 °C [1]. Previous studies have shown that the temperature gradient across most interior wall surfaces is minimal, making moisture transfer driven by thermal gradient negligible [2]. In addition, the thinness of these layers leads to minor internal temperature differences, allowing moisture transfer within them to be treated as an isothermal process [3,4,5]. Consequently, when investigating the moisture adsorption–desorption behavior of interior finishes, the effect of temperature on hygroscopic performance can be neglected [6,7]. Moreover, in modeling the moisture transfer within interior finishing layers, it is commonly assumed that the materials remain within the moisture-absorption range, allowing the neglect of liquid water transport [8]. Under these assumptions, water vapor diffusion serves as the dominant, and essentially exclusive, mechanism of moisture transfer between the indoor environment and the building envelope. The governing moisture transfer equation within interior finishing materials can be expressed as follows:

(1) $$\frac{\xi }{P_{\mathrm{sat}}}\frac{\partial P_{v}}{\partial t}=\frac{\partial }{\partial x}\delta_{v}\frac{\partial P_{v}}{\partial x}$$

where *ξ* is the moisture capacity (kg/m3); *P*_v_ is the water vapor partial pressure (Pa); *P*_sat_ is the saturated vapor pressure (Pa); *δ*_v_ is the water vapor permeability coefficient(kg/(m·s·Pa)); *t* is time(s); and *x* is the spatial coordinate along the thickness direction.

According to this equation, accurately determining the hygroscopic properties of interior finishing materials—particularly the moisture capacity and water vapor permeability coefficient—is crucial for accurately modeling moisture transfer between the building envelope and the indoor environment [9,10]. Although the moisture capacity has been extensively studied, the present work focuses primarily on the water vapor permeability coefficient.

In porous building materials, a complex network of pores and capillaries provides pathways for moisture migration. Driven by vapor pressure or concentration gradients, water vapor molecules diffuse from high-pressure (or high-concentration) regions to low-pressure (or low-concentration) ones. The water vapor permeability coefficient (*δ*_v_) quantifies this process and represents the ability of a porous material to transmit vapor. It is derived from Fick’s law in combination with the ideal gas law and is defined as the mass of water vapor (kg) passing through a 1 m thick layer per unit area in 1 s under a vapor pressure difference of 1 Pa between the two sides of the specimen, with units of kg/(m·s·Pa) [11]. Based on this definition, *δ*_v_ can be directly applied to calculate the vapor flux when diffusion is driven by a vapor pressure difference, making it widely used in engineering analysis. Among available testing techniques, the cup method specified in ISO 12570 [12] has been widely adopted for testing the water vapor permeability coefficient. This standard provides detailed descriptions for determining *δ*_v_. This method provides detailed testing procedures and environmental conditions but defines only five standardized temperature–humidity combinations.

Experimental studies conducted by various researchers using the cup method have shown that *δ*_v_ is affected by multiple parameters, and its relationship with environmental humidity differs substantially across materials. For example, Kuishan Li [13] employed the dry-cup method to determine the water vapor permeability coefficients for several building materials—including expanded polystyrene, extruded polystyrene, polyurethane, cement mortar, concrete, and porous clay brick—under external RH conditions ranging from 11.3% to 97.3%. Based on the test results, an empirical correlation between *δ*_v_ and external RH was established. However, the analysis was conducted with only six data points, and external RH alone could not accurately represent the actual material’s moisture state, limiting the accuracy of the derived function. Shuqin Chen and Yinyan Lv [14] investigated the vapor permeability of UV-aged engineered bamboo using the wet-cup method, with nine internal RH levels and an external environment of 25 °C and 50% RH. Their results indicated a power-law relationship between *δ*_v_ and moisture content, but large data scatter led to high uncertainty in the fitted parameters, highlighting the need for improved correction and fitting approaches. Oly Vololonirina and Bernard Perrin [15] studied the effects of air-layer thickness, material thickness, humidity gradient, and airflow velocity on *δ*_v_ for three insulation materials using the wet-cup method. They reported that the resistance of the internal air layer and airflow velocity significantly affected *δ*_v_, whereas specimen thickness and surface area had a limited impact. However, the study did not establish a quantitative relationship between *δ*_v_ and relative humidity. Similarly, T. Colinart and P. Glouannec [16] demonstrated that deviations between theoretical and actual internal RH conditions caused substantial errors—ranging from 5% to 450%—in the calculated vapor diffusion resistance factor (Sd). They proposed a correction approach to estimate the “true” Sd, but their focus was mainly on measurement error rather than the intrinsic variation in δv across different RH ranges. Further studies have investigated dry- and wet-cup conditions for various materials. Oly Vololonirina and Marie Coutand [17] investigated the water vapor diffusion resistance of wood materials using the cup method under two humidity conditions: internal RH of 9% and external RH of 50%, and internal RH of 97% and external RH of 50%. They conducted a comparative analysis to assess the influence of dry- and wet-cup methods on the measured results. Kazuma Fukui and Satoru Takada [18] measured the water vapor permeability of gypsum board, autoclaved aerated concrete, expanded polyurethane, calcium silicate board, ceramic tiles, plywood, and wood using the cup method. Based on Fick’s law of diffusion, they calculated the actual relative humidity within the materials and established approximate functions relating the water vapor permeability to the actual relative humidity of the materials. However, except for the gypsum board, their dataset included only 3–4 RH levels, leading to limited fitting accuracy. In addition, air-layer resistance was not explicitly excluded. Chi Feng [19] proposed that, due to the pronounced moisture hysteresis observed in most building materials, water vapor permeability should be characterized as a function of the material’s moisture content rather than relative humidity. Nevertheless, his experimental design employed only three humidity conditions, focusing primarily on the relationship between moisture content and water vapor permeability under different preconditioning states. Staf Roels [20] compiled the experimental procedures and results from six laboratories on the hygric properties of calcium silicate board, ceramic tiles, and porous concrete. The study noted that, in most current research, water vapor permeability is measured according to the ISO 12572; all laboratories except Laboratory 6 used the salt solution cup method. Comparison of the results revealed that, although measurements were consistent in order of magnitude across laboratories, the highest values were two to four times greater than the lowest, indicating considerable data variability. Furthermore, the study did not clarify whether the reported RH refers to the average RH or only to the RH of one side of the cup. Rasha Mustapha and Assaad Zoughai [21] briefly introduced the ASTM [22] method for measuring water vapor permeability, which involves only two humidity conditions: internal RH of 0% and external RH of 50%, and internal RH of 100% and external RH of 50%. They pointed out that this method is not suitable for materials with high water vapor permeability. Consequently, they proposed an improved approach that estimates the material’s water vapor permeability by separately estimating the air-layer resistance; however, for each material, only a single test condition was used to determine the water vapor permeability. Synthesizing the aforementioned studies, it is evident that, although the cup method standardized in ISO 12572 is widely employed for measuring water vapor permeability, current research still presents notable limitations, particularly regarding experimental humidity conditions.

In summary, although the cup method has been widely used, significant challenges remain: Limited humidity range—most studies tested only a few discrete RH points, making it difficult to derive continuous functional relationships; Inadequate microstructural interpretation—few studies have linked vapor permeability behavior to the pore structure and sorption characteristics of materials.

Gypsum-based inorganic mineral materials exhibit fast moisture absorption, low desorption hysteresis, and high humidity responsiveness due to their well-developed microporous structure, large specific surface area, and strong water adsorption capacity. Consequently, they are regarded as promising high-performance materials for indoor humidity regulation [23,24]. Such materials can passively buffer indoor humidity fluctuations through the adsorption and desorption of water vapor, thereby enhancing thermal comfort in living spaces and reducing building energy consumption. Among various inorganic mineral components, zeolite has attracted particular attention due to its regular, crystalline microporous channels and tunable pore structure. It not only possesses molecular sieve properties but also demonstrates excellent thermal and mechanical stability. Through ion exchange and structural modification, it can be functionalized to achieve specific adsorption and mass transfer characteristics [25,26,27]. Diatomite, a widely available and environmentally friendly porous siliceous material, features high porosity and strong adsorption capacity. Diatomite-based moisture-regulating materials can adjust indoor humidity without energy input, making them highly attractive in the field of sustainable building materials [28]. In recent years, with the development of diatomite-based plasters and composite building materials, diatomite has been extensively applied in interior finishes with humidity-regulating properties [29]. In addition, silicate minerals, composed of interconnected SiO4 tetrahedra, possess two-dimensional polymeric fragment structures and open channel features. Such structural characteristics endow these materials with high water adsorption capacity, conferring excellent potential for humidity control during moisture adsorption, desorption, and water vapor diffusion processes [30]. The correction factor method effectively predicts the water vapor permeability of earthen materials across wide ranges of relative humidity and suction [31]. Other researchers have demonstrated that the extended cup method allows a rigorous determination of both the mass diffusivity and the permeability for an inert solid phase by selecting suitable operating conditions based on the synthetic diagram [32].

Therefore, this study focuses on gypsum-based inorganic mineral materials, systematically investigating their water vapor transport characteristics under different environmental RH conditions. Using the cup method, the effects of the average RH and RH gradients on the water vapor permeability were quantitatively analyzed. A functional relationship between water vapor permeability and average RH was established, revealing its variation trend over an average RH range of 20–90%. Furthermore, the pore characteristics and water vapor transport properties of three representative inorganic mineral materials were analyzed at the microstructural level, providing theoretical insight and experimental evidence for a deeper understanding of the moisture transport mechanisms in gypsum-based composites and their humidity-regulating design in building interior applications.

## 2. Materials and Method

### 2.1. Materials

Gypsum was used as the base material, and diatomite, zeolite, and magnesium aluminum silicate were incorporated at a mass ratio of 2:1 (gypsum to inorganic mineral material). The water-to-solid ratio was set at 0.6. The mixtures were prepared to produce three types of gypsum-based inorganic mineral material specimens: gypsum–diatomite (G-D), gypsum–zeolite (G-Z), and gypsum–magnesium aluminum silicate (G-A). The densities of the four materials, including pure gypsum, G-D, G-Z, and G-A, were 1110, 865, 1150, and 1170 kg/m^3^, respectively. The chemical compositions of the materials are presented in Table 1.

**Figure 1:**
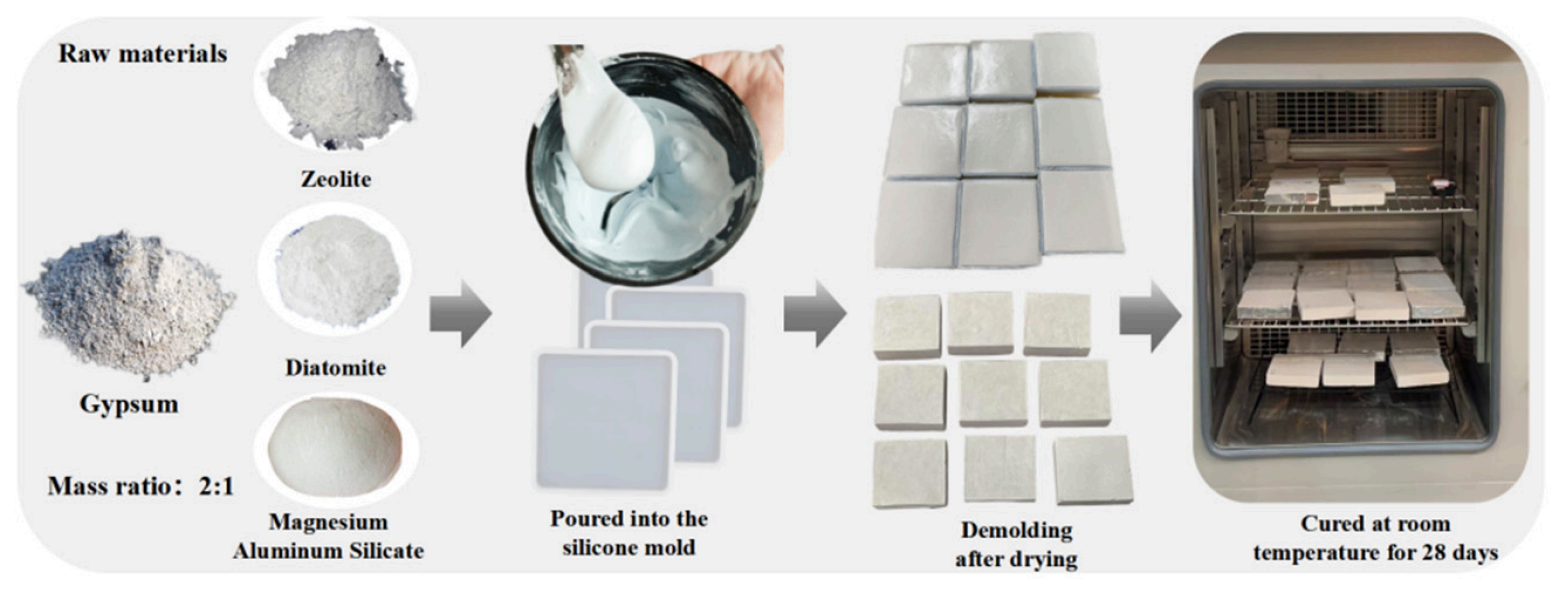
Fabrication process of gypsum-based inorganic mineral materials.

The detailed preparation procedure is illustrated in Figure 1. The specimens were cast using silicone molds measuring 10 cm × 10 cm × 2 cm. After drying and demolding, all samples were cured under ambient conditions for 28 days to ensure stabilization of their internal structure.

### 2.2. Experimental Methods

In this study, the water vapor permeability of the materials was determined using the cup method specified in accordance with ISO 12572 [12]. According to T. Colinart [16], experimental observations indicate that the internal RH is difficult to stabilize under the dry-cup condition. Therefore, the wet-cup method was adopted in this work. The cured specimens were fixed to the lids of plastic containers (13.5 cm × 13.5 cm × 7 cm) using epoxy adhesive. Each lid was manually cut with a square opening slightly larger than 10 cm × 10 cm to accommodate the specimen. After the epoxy resin was fully cured, the specimen lid assemblies were dried in an oven at 40 °C and then preconditioned at 23 °C under an RH corresponding to the target testing condition (i.e., the low-humidity environment inside the cup). Measurements were initiated immediately after preconditioning.

**Figure 2:**
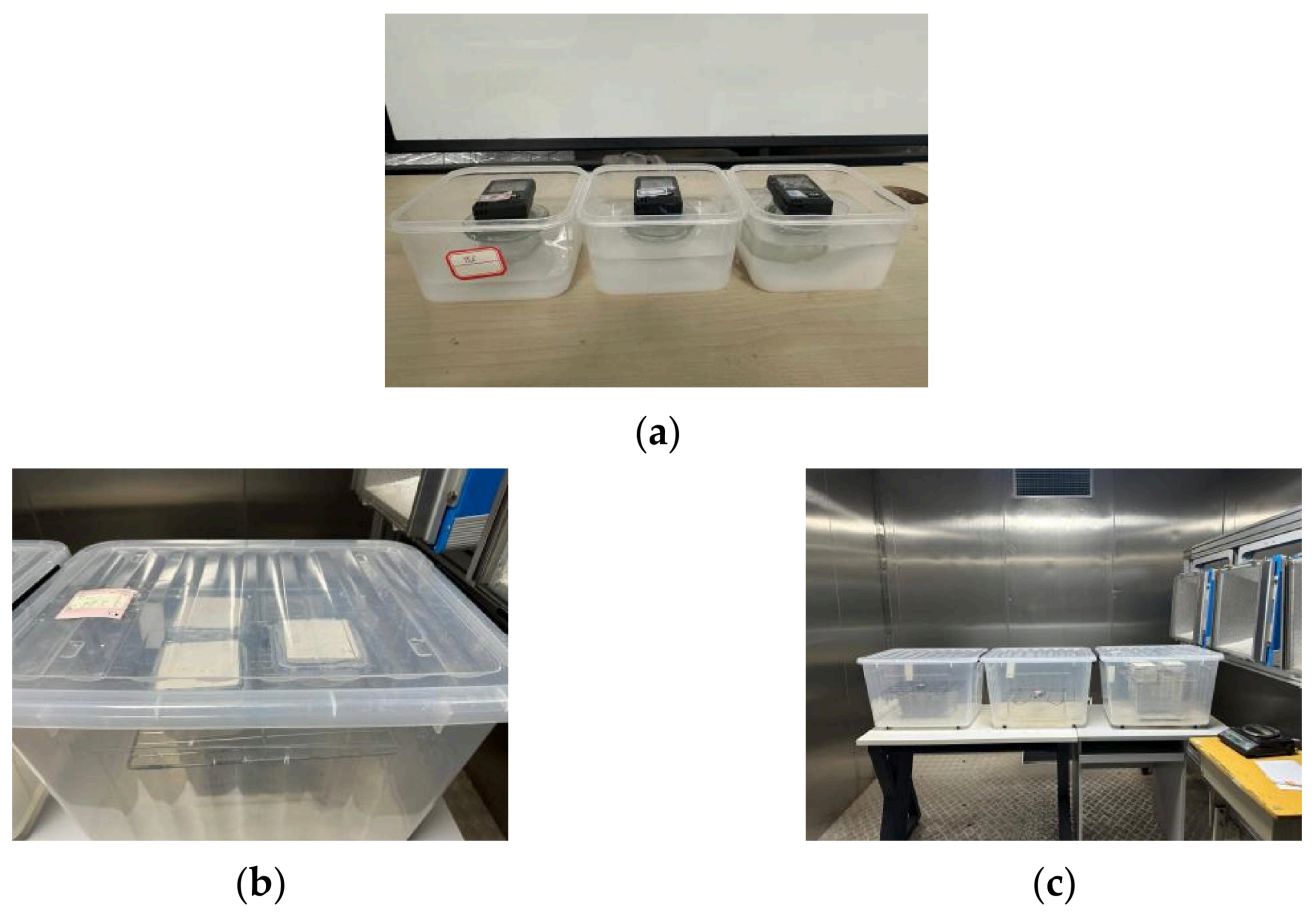
Experimental apparatus scheme. (**a**) Wet cup apparatus; (**b**) Single-condition test; (**c**) Multi-condition test.

In this study, the coefficient of variation (CV) was used to assess the consistency of the water vapor permeability coefficients obtained from three parallel specimens under each test condition, as shown in Equation (2):

(2) $$\mathrm{CV}=\frac{s}{\bar{x}}\times 100\%$$

where *CV* is the coefficient of variation (%); *s* is the sample’s standard deviation; and $\bar{x}$ is the sample mean. Considering the sensitivity of the water vapor permeability test to experimental variability, a relatively strict criterion was adopted. When the CV was less than 5%, the results were considered sufficiently stable and reproducible. When the CV was equal to or greater than 5%, the results were considered insufficiently stable, and the corresponding test was repeated in accordance with the experimental procedure until the required reproducibility was achieved. The internal and external RH levels were maintained using saturated salt solutions, as listed in Table 2. The assembled specimen–cup units were placed inside a larger sealed chamber. Temperature and humidity sensors were mounted on both sides of the specimen using supporting racks to monitor external humidity continuously. The concentration of the salt solutions was adjusted as necessary to maintain stable humidity conditions both inside and outside the cup. The experimental setup is illustrated in Figure 2.

Before each experiment, the specimens were preconditioned under the designated environmental conditions. During the test, the total mass of the specimen together with the sealed container was recorded at 24 h intervals. The test was considered complete when seven consecutive measurements exhibited a linear increase in mass over time. *A* scatter plot of total mass versus time was then plotted, and the slope of the fitted linear regression line was defined as the mass change rate, *M* (kg/s). The moisture flux per unit area was subsequently calculated according to Equation (3):

(3) $$g_{v}=\frac{M}{A}$$

(4) $$\delta_{v}=\frac{g_{v}\cdot d_{\mathrm{mat}}}{\Delta P_{v}}$$

where *g*_v_ is the moisture flux density, kg/(m^2^·s); *M* is the mass change rate, kg/s; *A* is the specimen area, m^2^; *δ*_v_ is the total: measured water vapor permeability coefficient, kg/(m·s·Pa); *P*_v_ is the water vapor partial pressure, Pa; and *d*_mat_ is the thickness of the material.

**Figure 3:**
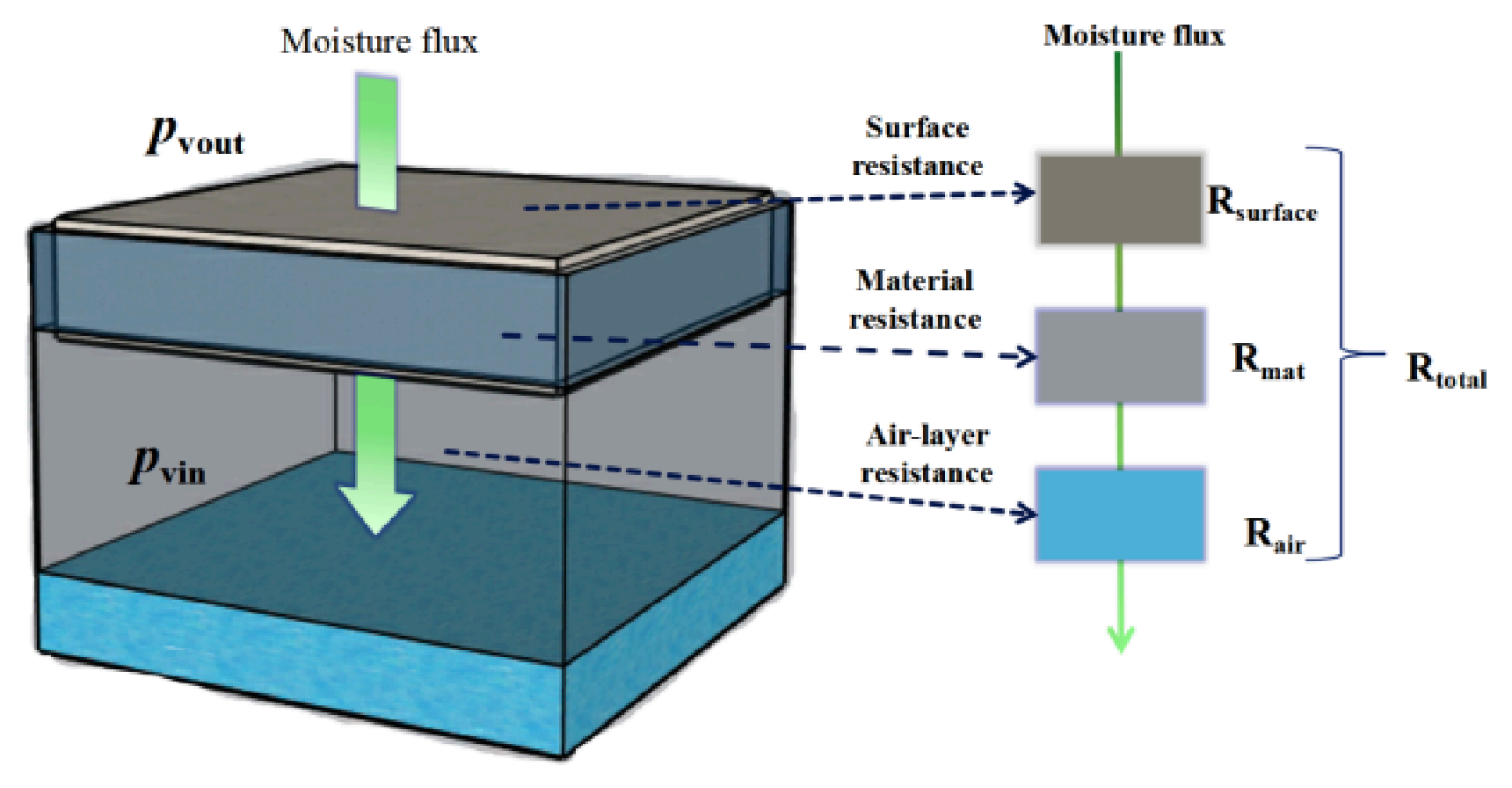
Schematic diagram of water vapor resistance.

In this testing method, the water vapor transfer from the high-humidity side to the low-humidity side passes through three sequential vapor resistance components, as illustrated in Figure 3: (1) the external surface resistance of the material, (2) the intrinsic material resistance, and (3) the air-layer resistance inside the cup [16].

Accordingly, the total moisture flux of the system can be expressed as

(5) $$g_{v}=\frac{p_{\mathrm{vout}}-p_{\mathrm{vin}}}{R_{\mathrm{surface}}+R_{\mathrm{mat}}+R_{\mathrm{air}}}=\frac{p_{\mathrm{vout}}-p_{\mathrm{vin}}}{R_{\mathrm{total}}}$$

(6) $$R_{\mathrm{mat}}=\frac{d_{\mathrm{mat}}}{\delta_{v,\mathrm{mat}}}$$

(7) $$R_{\mathrm{surface}}=\frac{1}{h_{m}}$$

(8) $$R_{\mathrm{air}}=\frac{d_{\mathrm{air}}}{\delta_{\mathrm{air}}}$$

(9) $$\delta_{v,\mathrm{mat}}=\frac{\delta_{v,\mathrm{total}}}{1-\frac{\delta_{v,\mathrm{total}}}{d_{\mathrm{mat}}}(\frac{1}{h_{m}}+\frac{d_{\mathrm{air}}}{\delta_{v,\mathrm{air}}})}$$

where *P*_vout_ is the water vapor partial pressure outside the cup, Pa; *P*_vin_ is the water vapor partial pressure inside the cup, Pa; R_surface_ is the surface diffusive vapor resistance, m/s; *h*_m_ is the vapor mass exchange coefficient, s/m; R_mat_ is the material diffusive vapor resistance, m/s; *d*_mat_ is the thickness of material, m; *δ*_v,mat_ is the intrinsic material water vapor permeability, kg/(m·s·Pa); R_air_ is the air-layer diffusive vapor resistance, m/s; d_air_ is the thickness of the air-layer, m; and *δ*_v,air_ is the water vapor permeability coefficient of the air-layer, kg/(m·s·Pa).

According to the calculation procedure in ISO 12572 (Equation (2)), the measured vapor permeability (*δ*_v_) represents the overall permeability coefficient of the cup–specimen system. Therefore, in this study, corrections were applied to account for the air-layer resistance and the surface vapor resistance in order to obtain the intrinsic water vapor permeability coefficient of the material.

### 2.3. Test Condition Setup

#### 2.3.1. RH Gradients at the Same Average RH

To investigate how different RH gradients affect the water vapor permeability of materials under the same average relative humidity, four sets of average relative humidity test conditions were designed in Table 3, at an average RH of approximately 59% (Cases 3 and 4) and 64% (Cases 5 and 6). Due to the limited precision of saturated salt solution control, minor deviations in average RH occurred. To enhance data continuity, two additional cases with average RH values of 43% (Case 1) and 45% (Case 2) were included; because their difference is small, they are treated as comparable. Similarly, cases with average RH values of 69% (Case 7) and 71% (Case 8) are also regarded as equivalent.

#### 2.3.2. Different Average RH at the Same RH Gradient

To further investigate the influence of average RH, test conditions were designed with the same RH gradient but different average RH values. For an RH gradient of 10%, three average RH conditions were established (Cases 1–3), while for gradients of 32%, 43%, and 52%, two average RH conditions were established for each. The specific RH conditions are summarized in Table 4.

#### 2.3.3. Relationship Between Water Vapor Permeability and Average Relative Humidity

To systematically investigate the variation in water vapor permeability with average RH, this study further designed 19 experimental conditions spanning a wide average RH range (22–90%) The specific experimental settings are summarized in Table 5.

## 3. Results

### 3.1. Influence of RH Gradients

**Figure 4:**
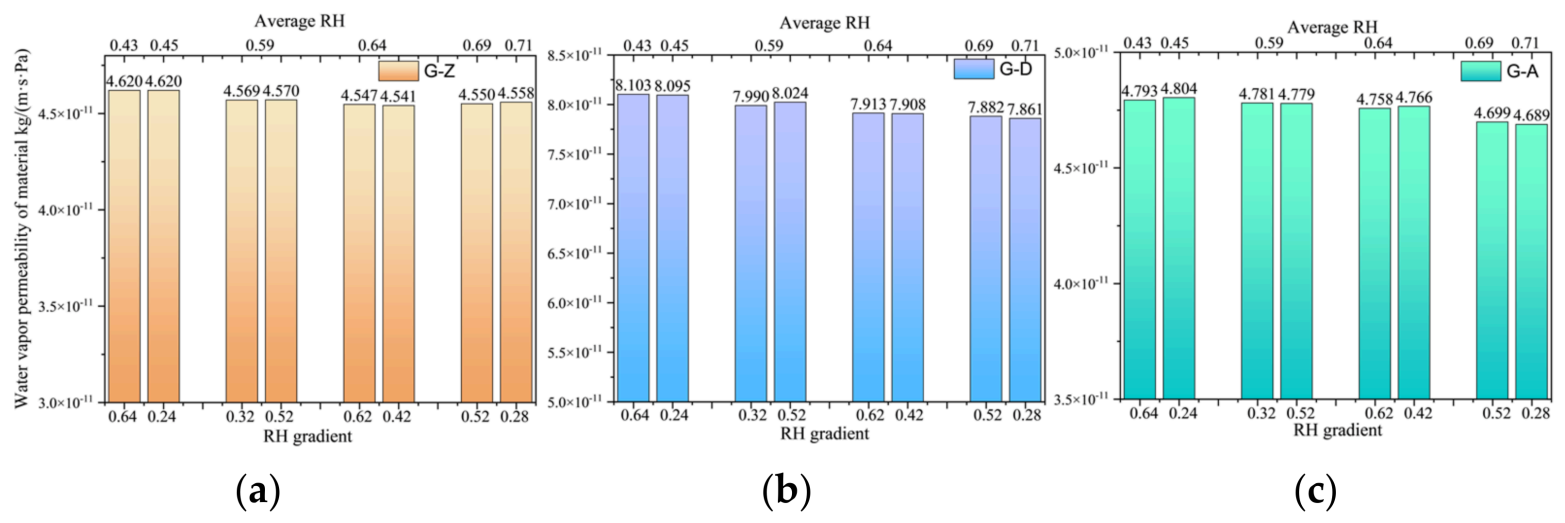
Effect of relative humidity gradient on the water vapor permeability of materials. (**a**) G-Z, (**b**) G-D, and (**c**) G-A.

When the RH gradient within the specimens is varied while maintaining a constant average RH (Figure 4), the water vapor permeability remains nearly unchanged. The values of the water vapor permeability coefficient under each condition are indicated in the figure (note that all values in Figure 4 should be multiplied by 10^−11^), and the differences are all less than 1%. This indicates that, within the tested conditions, the RH gradient does not constitute a dominant factor governing the water vapor transport capacity of the materials.

### 3.2. Influence of the Average RH

**Figure 5:**
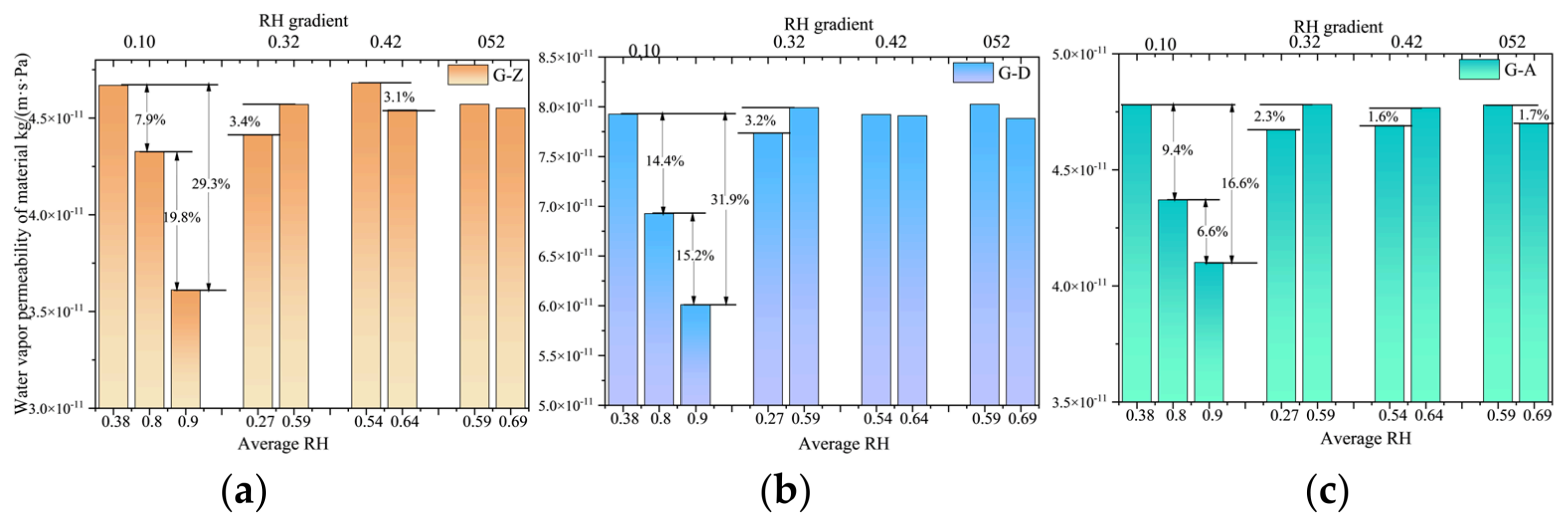
Effect of the average relative humidity on the water vapor permeability of materials. (**a**) G-Z, (**b**) G-D, and (**c**) G-A.

As illustrated in Figure 5, when the RH gradient is held constant, variations in the average RH lead to pronounced changes in the water vapor permeability of the materials. This effect becomes especially significant at high average RH levels, where the water vapor permeability coefficient is highly sensitive to changes in average RH. For example, under an RH gradient of 32%, the water vapor permeability of the three materials at an average RH of 59% differs by only 3.4%, 3.2%, and 2.3%, respectively, compared to that at an average RH of 27%. In contrast, under the RH gradient of 10%, the water vapor permeability at an average RH of 90% decreases markedly by 29.3%, 31.9%, and 16.6% relative to that at an average RH of 38%. The differences observed under other test conditions are also indicated in Figure 5. These findings clearly indicate that the average RH exerts a significant and non-negligible influence on the water vapor permeability of the materials, highlighting the critical role of ambient moisture conditions in evaluating material hygrothermal performance.

In summary, the water vapor permeability of the materials exhibits low sensitivity to RH gradient but high sensitivity to variations in average RH. This finding highlights that, when predicting or modeling vapor diffusion in building materials, the mean environmental humidity plays a more critical role than the instantaneous RH difference across the material layer.

### 3.3. Relationship Between Water Vapor Permeability and Average RH

**Figure 6:**
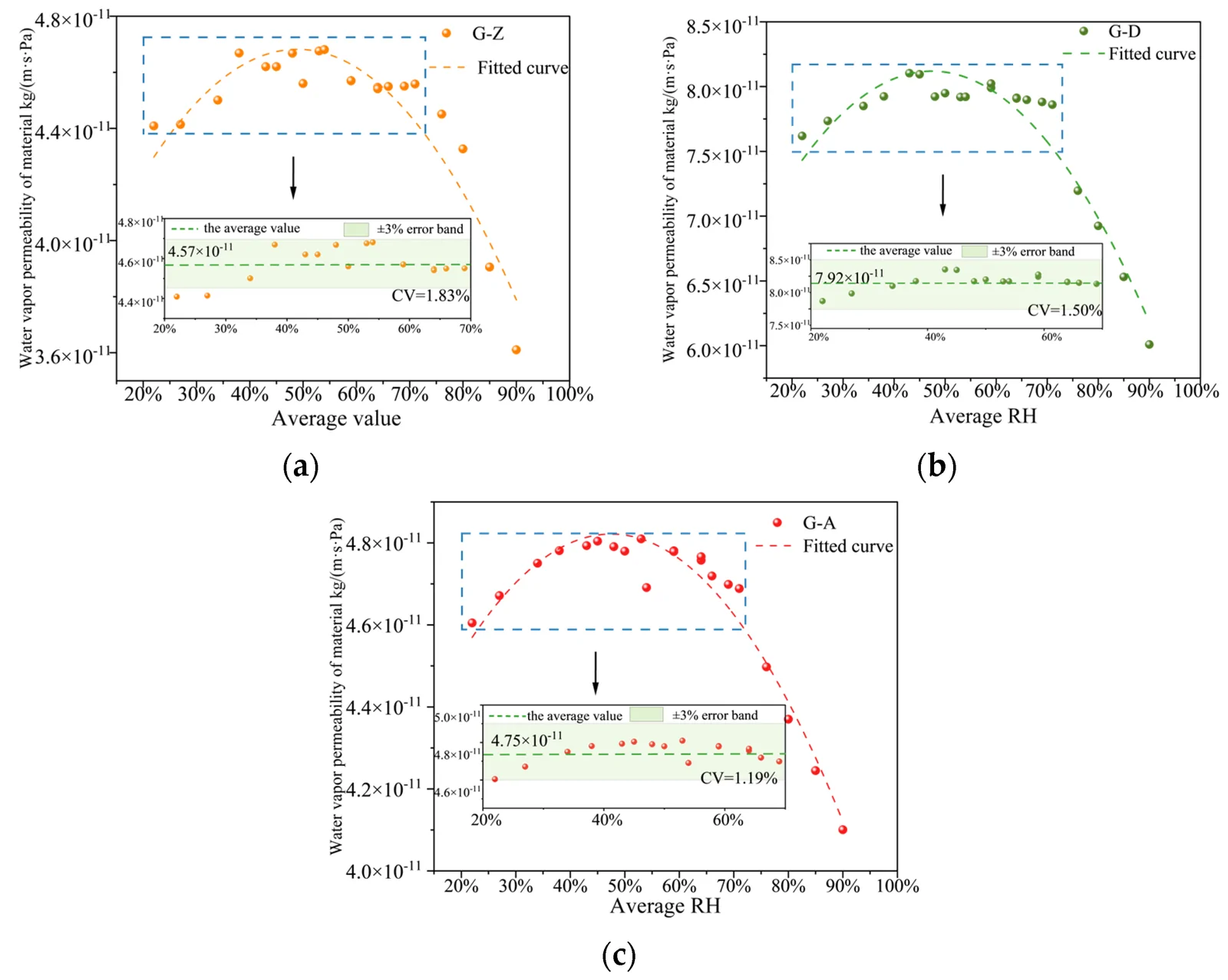
The relationship between the water vapor permeability coefficient and average relative humidity of the materials. (**a**) G-Z, (**b**) G-D, and (**c**) G-A.

Taking the average RH as the horizontal axis, the relationship between the water vapor permeability coefficient and the average RH was plotted, as shown in Figure 6.

As shown in Figure 6, the water vapor permeability coefficient of the three materials exhibits a distinct non-monotonic trend with increasing average RH—first increasing slightly and then decreasing significantly. When the average RH is below approximately 70%, the permeability coefficient shows a gradual upward tendency. This behavior can be attributed to the dominance of vapor-phase diffusion through the pore network under low-humidity conditions. As RH increases, the adsorbed water layer on the pore surfaces thickens, enhancing the local concentration gradient of water vapor molecules and thereby promoting diffusion.

According to the Kelvin equation, capillary condensation can occur within nanoscale pores of porous materials at relative humidity levels below 100%. In general, the smaller the pore size, the lower the critical relative humidity required for capillary condensation to occur [33]. Considering the pore size distribution of the materials investigated in this study, when the relative humidity exceeds 70%, the fine pores within the materials may meet the conditions for capillary condensation, leading to the formation of liquid water within these pores. The resulting liquid water may partially obstruct the pore pathways and consequently reduce the water vapor permeability of the materials.

Additionally, as moisture accumulates, the internal RH gradient within the material diminishes, reducing the driving potential for vapor diffusion. The combined effects of capillary condensation and weakened diffusion potential ultimately result in a pronounced decrease in the overall water vapor permeability.

A further analysis of the permeability coefficients within the 22–70% average RH range shows that the coefficient of variation (CV) of the measured data is very small—1.82% for G–Z, 1.50% for G–A, and 1.18% for G–D—indicating excellent data stability and repeatability. Therefore, the mean value within this RH range can be considered the representative water vapor permeability coefficient for each material. As illustrated in the enlarged view of Figure 6, an error band corresponding to the mean value ±3% encompasses all data points, confirming that under moderate humidity conditions (i.e., when the average indoor–outdoor RH is below 70%), the water vapor permeability of the materials can reasonably be regarded as constant.

The overall variation in the water vapor permeability coefficient of the three materials with the average RH (22–90%) can be well described by a quadratic polynomial function, as summarized in Table 6. The fitting accuracy of the quadratic polynomial model is satisfactory for all three materials. The coefficients of determination (R^2^) are 0.88, 0.95, and 0.93 for G–Z, G–A, and G–D, respectively, indicating that the model explains more than 88% of the variation in the experimental data. Meanwhile, the corresponding residual sum of squares (RSS) values are all very small, further confirming the close agreement between the fitted and measured data. These results demonstrate that the quadratic polynomial function provides an accurate and reliable representation of the relationship between the water vapor permeability coefficient and the average relative humidity for the studied gypsum-based materials.

## 4. Discussion

As shown in Figure 6, distinct differences in water vapor permeability coefficients were observed among the three gypsum-based composite materials under identical test conditions. The G-D composite exhibited a markedly higher water vapor permeability coefficient than G-Z and G-A, while the latter two presented similar yet relatively lower values. These variations suggest that differences in pore structure and connectivity play a decisive role in controlling vapor transport.

In combination with the sorption isotherm results [34], the hygroscopic and vapor transport characteristics of the three materials were further interpreted from a microstructural perspective, as summarized in Table 7. The G-Z composite is characterized by numerous fine pores with a high specific surface area and a partially closed pore network, resulting in strong moisture adsorption but limited water vapor diffusion. The G-D composite contains abundant mesopores with continuous and well-connected diffusion pathways, enabling moderate adsorption and highly efficient vapor transport. In contrast, the G-A composite exhibits large and tortuous pores, leading to weak adsorption capacity but a continuous, albeit resistance-prone, diffusion network.

**Figure 7:**
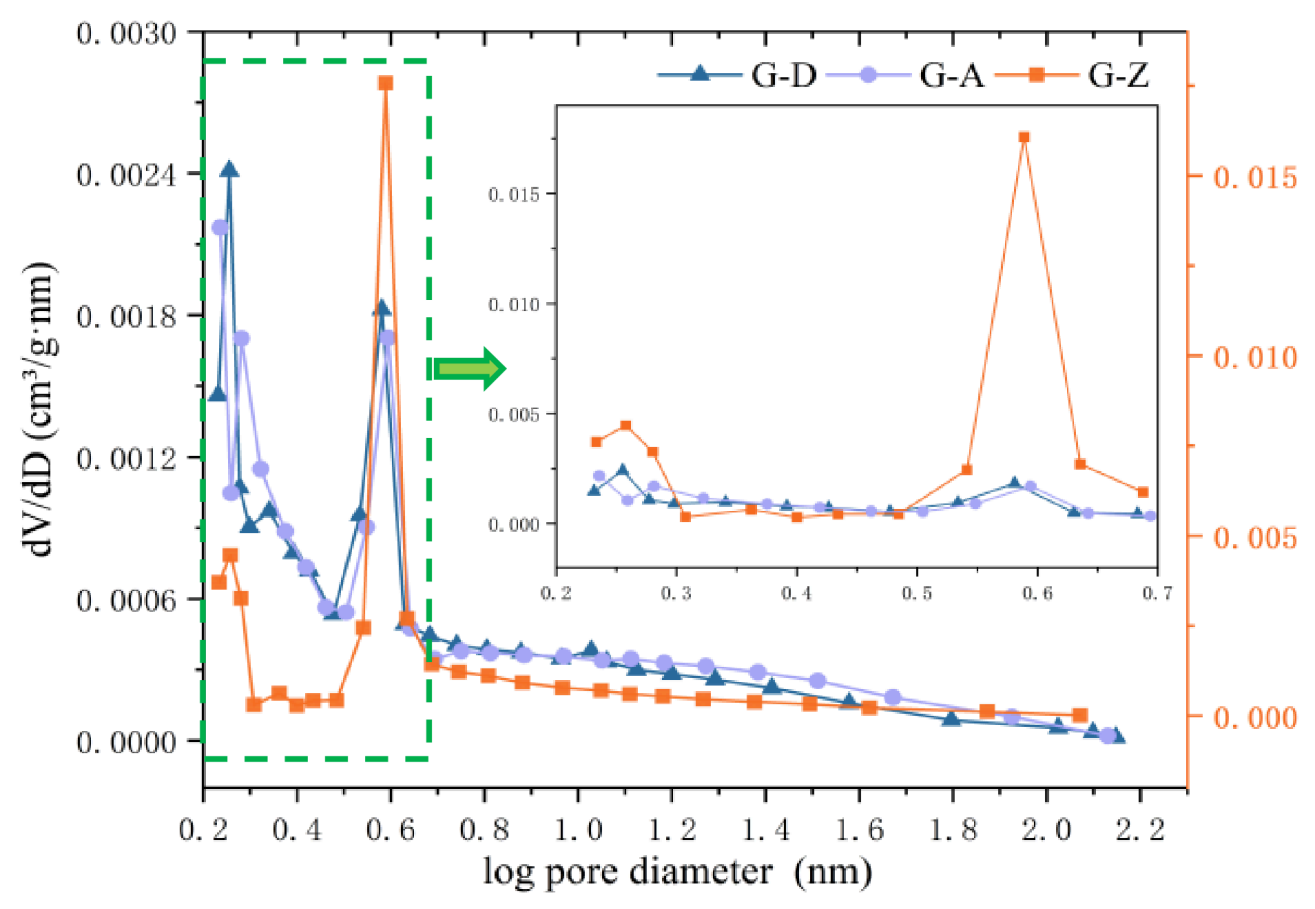
Pore size distribution of the materials.

Quantitative pore characteristics—including average pore size, specific surface area, and cumulative pore volume—are summarized in Table 8, and the corresponding pore size distributions are illustrated in Figure 7.

The G-Z composite has the smallest average pore size but the largest specific surface area and cumulative pore volume. This structure indicates that zeolite particles are mainly attached to the gypsum matrix, forming a highly developed microstructure dominated by micropores and small mesopores (0–5 nm). Such fine and dense pores enhance water vapor adsorption and storage but restrict pore connectivity, thereby limiting vapor diffusion and resulting in a relatively low water vapor permeability coefficient.

The G-A composite, by contrast, exhibits the largest average pore size—exceeding that of pure gypsum—while maintaining comparable specific surface area and cumulative pore volume. This indicates that the incorporation of magnesium aluminum silicate does not significantly enhance moisture storage capacity. Although its larger mesopores are theoretically favorable for diffusion, the complex, tortuous pore network increases diffusion resistance, leading to relatively low measured vapor permeability.

The G-D composite predominantly contains mesopores with moderate average size and a smaller proportion of micropores, resulting in lower specific surface area but good pore connectivity. Such a structure ensures continuous diffusion pathways and reduced resistance to vapor flow, thereby yielding superior water vapor permeability.

**Figure 8:**
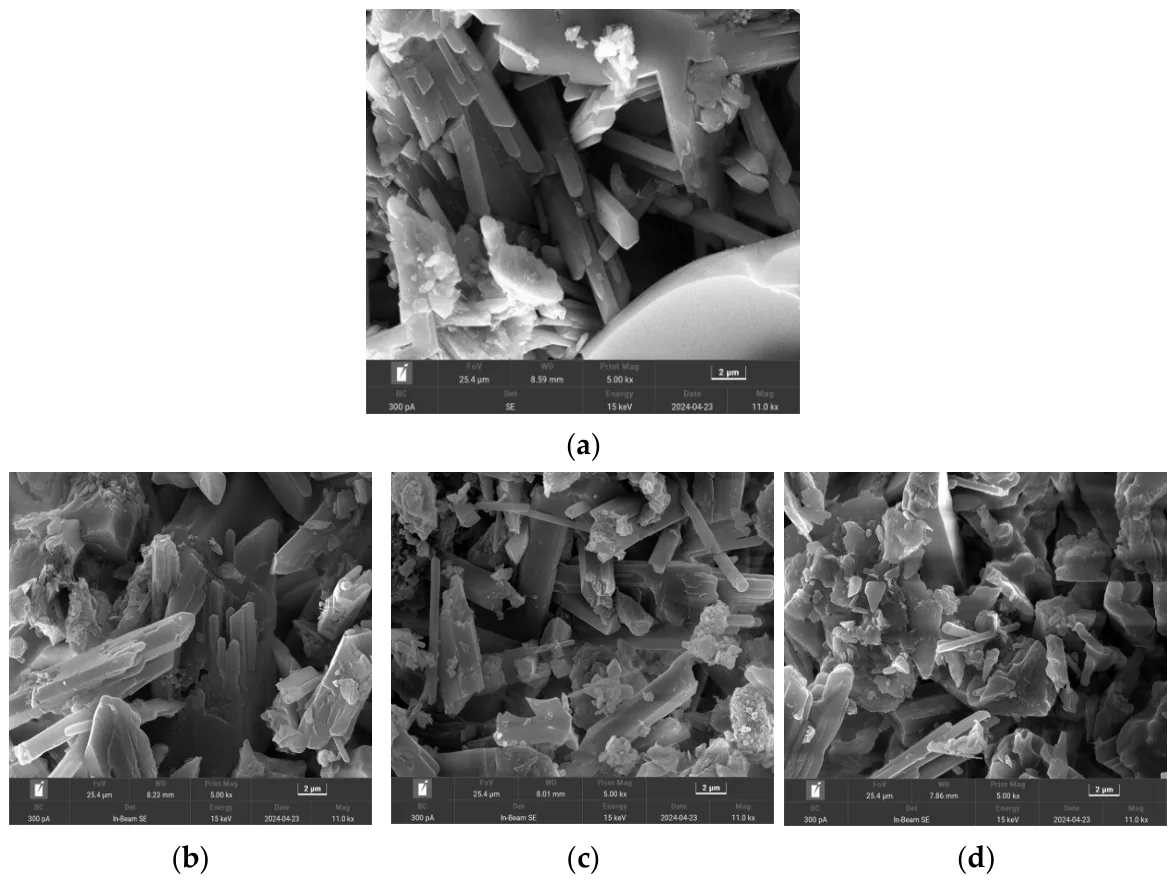
Scanning electron microscopy (SEM) images of the materials. (**a**) Gypsum, (**b**) G-Z, (**c**) G-A, and (**d**) G-D.

**Figure 9:**
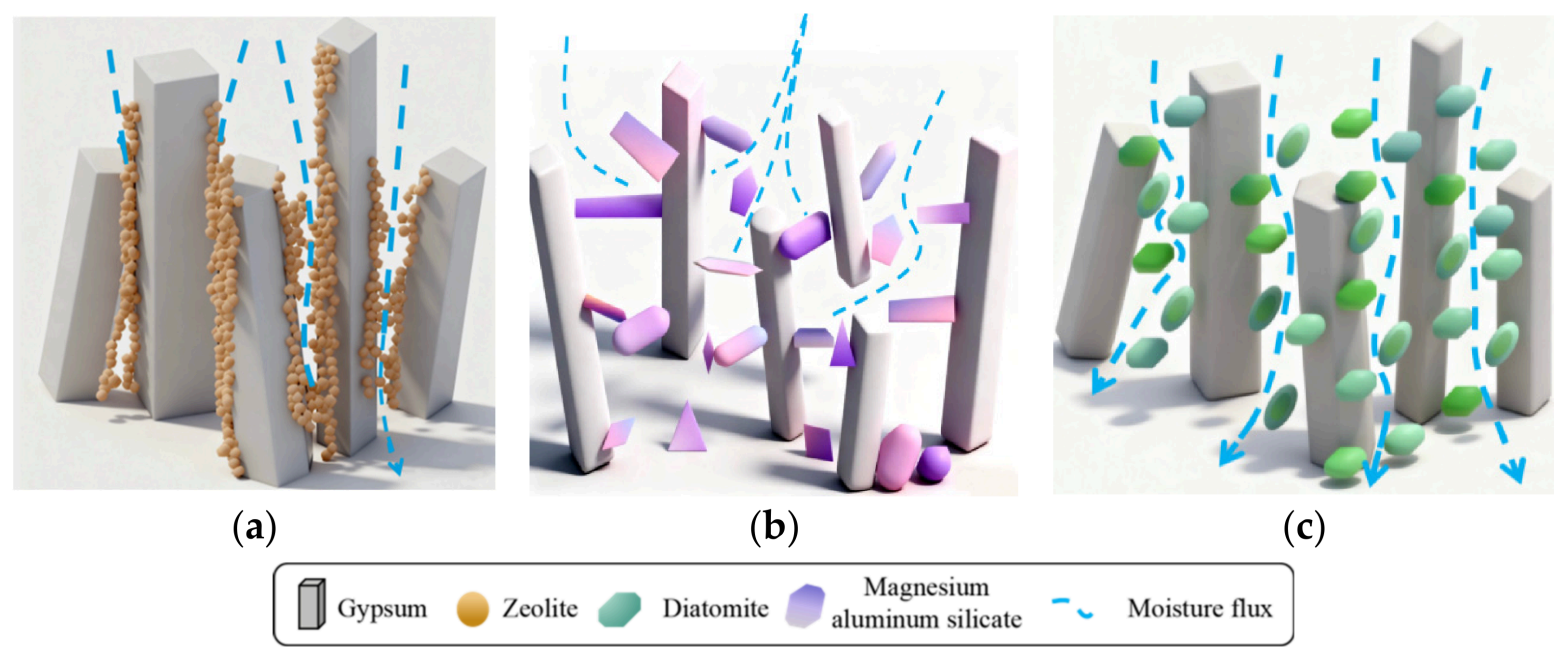
Schematic illustration of water vapor flow within the materials. (**a**) G-Z, (**b**) G-A, and (**c**) G-D.

To further illustrate the pore morphology and water vapor transport pathways of the three materials, scanning electron microscopy (SEM) was employed to observe their internal microstructures, as shown in Figure 8. Based on SEM observations and pore-size distribution analysis, schematic diagrams illustrating the water vapor diffusion behavior within the composites were developed (Figure 9). It should be noted that the primary purpose of Figure 9 is to qualitatively highlight the overall changes in pore morphology induced by the incorporation of different fillers. Therefore, only simplified representations of partially aligned gypsum crystals and the spatial distribution of additives within the gypsum matrix are shown, while other regions are omitted for clarity. This schematic provides an intuitive visualization of the dominant water vapor transport pathways in each composite system.

As observed in the SEM images, pure gypsum consists of interwoven, stacked columnar crystals that form a continuous solid framework enclosing pores of various sizes, resulting in a loose, porous overall morphology. Upon the addition of zeolite, granular zeolite particles predominantly attach to the surfaces of the columnar gypsum crystals, narrowing the intercrystalline gaps while increasing the total pore number and specific surface area. However, partial pore blockage occurs, which reduces pore connectivity and hinders vapor transport between adjacent gypsum columns. The incorporation of magnesium aluminum silicate results in the formation of dense, embedded structures within the gypsum framework, enlarging individual pores but simultaneously creating tortuous and poorly connected diffusion pathways that increase vapor transport resistance. In contrast, the diatomite particles are uniformly dispersed within the gypsum matrix, forming a relatively open structure with abundant interconnected meso- and macropores that facilitate continuous vapor diffusion channels.

In summary, hygroscopic behavior is primarily governed by micropore abundance, specific surface area, and cumulative pore volume. In contrast, the water vapor permeability coefficient is mainly controlled by pore-size distribution and interconnectivity. The G-Z composite exhibits strong moisture adsorption due to its high specific surface area and numerous micropores, but pore blockage limits its permeability. The G-D composite, characterized by well-connected meso- and macropores, demonstrates relatively high vapor permeability. Although the G-A composite has a comparable moisture sorption capacity to G–D, its dense, embedded structure leads to limited pore connectivity and, consequently, lower permeability, similar to the behavior observed for G-Z. It should be noted that the above analysis is primarily qualitative and is based on pore-related parameters and SEM observations. Furthermore, the relative humidity gradients analyzed were not systematically distributed across the entire range of relative humidity. Further quantitative analysis of the relationship between pore characteristics, pore connectivity, and moisture transport properties is warranted in future studies.

## 5. Conclusions

- Among the humidity parameters, the RH gradient across the cup had a comparatively limited influence, whereas the average RH appeared to play a dominant role in determining the water vapor transport behavior under the tested conditions.
- A clear nonlinear dependence of water vapor permeability on average RH was observed. Below approximately 70% RH, the permeability increased slightly with rising RH, attributed to enhanced vapor-phase diffusion. The mean value within this RH range can be considered the representative water vapor permeability coefficient for each material.
- When the average RH exceeded 70%, the permeability dropped sharply due to capillary condensation and reduced pore connectivity. The overall variation across the full RH range (22–90%) was accurately described by a quadratic polynomial function.
- From a microstructural standpoint, the vapor transport and moisture adsorption characteristics of the materials are closely associated with their pore features. The G-Z composite, with abundant micropores and high specific surface area, exhibits strong hygroscopicity but limited vapor permeability due to pore blockage. The G-D composite features well-connected meso- and macropores that facilitate efficient vapor diffusion, resulting in high permeability but moderate hygroscopicity. In contrast, the G-A composite forms dense embedded structures that reduce pore connectivity and increase pathway tortuosity, resulting in low vapor permeability and adsorption capacity.

Overall, these findings provide a quantitative and mechanistic understanding of water vapor transport in gypsum-based materials. The results can serve as a theoretical and experimental basis for optimizing the design and application of humidity-regulating gypsum composites for energy-efficient, moisture-controlled building envelopes.

## Figures and Tables

**Table 1:** Chemical compositions of raw materials.

Element% Materials	C	O	Na	Mg	S	Si	Al	Ca	Fe	K	Ti	Cl
Gypsum	41.71	33.69	0.00	3.29	5.99	2.57	0.38	11.84	0.29	0.24	0.00	0.00
G-D	47.68	33.22	0.50	0.00	0.22	17.7	0.27	0.00	0.43	0.00	0.00	0.00
G-Z	54.03	31.46	0.18	0.11	0.23	10.27	1.93	0.58	0.36	0.86	0.00	0.00
G-A	8.71	41.98	1.26	1.35	9.40	12.26	8.94	14.27	1.12	0.58	0.40	1.25

**Table 2:** Relative humidity corresponding to salt solutions.

Saturated Salt Solutions	Relative Humidity
LiCl	11.3%
MgCl_2_	33.1%
K_2_CO_3_	43.2%
NaBr	57.6%
NaCl	75.3%
KCl	84.3%
Na_2_SO_4_	97.3%

**Table 3:** Experimental conditions with the same average RH but different RH gradients.

Average RH RH Conditions	43%	45%	59%		64%		69%	71%
Case	1	2	3	4	5	6	7	8
Internal	11%	33%	43%	33%	33%	43%	43%	57%
External	75%	57%	75%	85%	95%	85%	95%	85%
Gradient	64%	24%	32%	52%	62%	42%	52%	28%

**Table 4:** Experimental conditions with the same RH gradient but different average RH.

RH Gradient RH Conditions	10%			32%		42%		52%	
Case	1	2	3	4	5	6	7	8	9
Internal	33%	75%	85%	11%	43%	33%	43%	33%	43%
External	43%	85%	95%	43%	75%	75%	85%	85%	95%
Average	38%	80%	90%	27%	59%	54%	64%	59%	69%

**Table 5:** Experimental conditions.

Average (%)	22	27	34	38	43	45	48	50	53	54	59	59	64	64	66	69	71	76	80	85	90
Internal (%)	11	11	11	33	11	33	11	43	11	33	33	43	33	43	57	43	57	57	75	75	85
External (%)	33	43	57	43	75	57	85	57	95	75	85	75	95	85	75	95	85	95	85	95	95

**Table 6:** Quadratic polynomial fitting results of water vapor permeability coefficient (average RH range: 22–90%).

Material Parameters Fitting Function	y = a·x + b·x^2^ + c				
	a	b	c	R^2^	RSS
G-Z	5.19 × 10^−11^	−5.30 × 10^−11^	3.41 × 10^−11^	0.88	1.60 × 10^−23^
G-A	3.68 × 10^−11^	−3.88 × 10^−11^	3.95 × 10^−11^	0.95	3.61 × 10^−24^
G-D	1.01 × 10^−10^	−1.07 × 10^−10^	5.72 × 10^−11^	0.93	4.33 × 10^−23^

**Table 7:** Hygroscopic and water vapor permeability classification of the three materials.

Material	Sorption Capacity [31]	Vapor Permeability	Microstructural Features
G-Z	high	low	Small and abundant pores with high specific surface area and closed pore structure
G-D	low	high	Abundant mesopores with connected gas-phase pathways
G-A	low	low	Large pores with tortuous pathways

**Table 8:** Pore structure characteristics of the materials.

Material Microscopic Structure	Gypsum	G-Z	G-A	G-D
Average pore size (nm)	15.6933	9.6882	15.9216	12.0554
Specific surface area (m^2^/g)	9.0274	16.5814	7.1205	6.8705
Cumulative pore volume (cm^3^/g)	0.025884	0.042099	0.026674	0.020195

## Data Availability

The original contributions presented in this study are included in the article. Further inquiries can be directed to the corresponding author.

## Abbreviations

The following abbreviations are used in this manuscript:

G-Z	gypsum–zeolite
G-D	gypsum–diatomite
G-A	gypsum–magnesium aluminum silicate
RH	relative humidity
